# Three Visual–Diagnostic Methods for the Detection of Enamel Cracks: An In Vitro Study

**DOI:** 10.3390/jcm12030973

**Published:** 2023-01-27

**Authors:** Tim Hausdörfer, Lisa Harms, Philipp Kanzow, Michael Hülsmann

**Affiliations:** 1 Department of Preventive Dentistry, Periodontology and Cariology, University Medical Center Göttingen, D-37075 Göttingen, Germany; 2 Clinic for Preventive Dentistry, Periodontology and Cariology, Center of Dental Medicine, University of Zurich, CH-8032 Zurich, Switzerland

**Keywords:** cracked tooth syndrome, tooth fractures, diagnostic errors, dental operating microscope, enamel crack, fiber-optic transillumination, near-infrared transillumination

## Abstract

Tooth fractures are a common cause of tooth loss, frequently starting as enamel cracks. However, methods for the detection of enamel cracks are poorly investigated. The aim of the study was the validation of three clinical methods for the detection of enamel cracks: dental operating microscope (DOM), near-infrared transillumination (NIR), and fiber-optic transillumination (FOTI), with hard-tissue slices serving as controls. A total of 89 extracted teeth, set up as diagnostic models, were investigated, and the maximum crack depth was scored by two examiners. The actual crack depth was determined microscopically (25×) using horizontal sections. The accuracy of each method was analyzed using receiver operating characteristic (ROC) curves. Across all tooth surfaces, the area under the curve (AUC) amounted to 0.57 (DOM), 0.70 (FOTI), and 0.67 (NIR). For crack detection on vestibular/oral surfaces, the AUC was 0.61 (DOM), 0.78 (FOTI), and 0.74 (NIR); for proximal surfaces, it was 0.59 (DOM), 0.65 (FOTI), and 0.67 (NIR). However, the actual crack depth was underestimated with each method (*p* < 0.001). Under in vitro conditions, FOTI and NIR are suitable for detection of enamel cracks, especially on vestibular and oral tooth surfaces. However, an exact estimation of crack depth is not possible. Therefore, FOTI and NIR seem to be helpful for the clinical detection of enamel cracks.

## 1. Introduction

Tooth fractures frequently develop from incomplete fractures or cracks that extend through the enamel into the dentin [1]. The most recent classification of fractures by the American Association of Endodontists categorizes fracture formation and progression into five groups: (1) craze line, (2) cuspal fracture (3), cracked tooth, (4) split tooth, and (5) vertical root fracture [2]. Initial tooth cracks may show a progressive course. First, a craze line develops, i.e., an initial lesion running parallel to the prismatic arrangement of the enamel crystals. It may grow longitudinally and spread into the dentin [3]. In view of the wide variety of forms, a variety of symptoms can also occur, which are often associated with pain stimuli on mastication and cold [4]. The pulp and/or the periodontal tissue might be damaged as a result of bacterial leakage [5,6,7] with accompanying symptoms of an irreversible pulpitis, pulp necrosis, or periapical periodontitis [8], and some cracks may finally result in complete tooth fractures with or without complete separation of the fragments [9].

In the early stages of a tooth fracture, diagnosis is difficult because the tooth usually is asymptomatic and the crack cannot be seen without disclosing diagnostic tools [10]. As outlined above, it is important to detect cracks at an early stage to avoid further damage such as irreversible pulpitis, pulp necrosis, or even tooth loss [11].

Commonly used methods for diagnosing cracks include transillumination, magnification with a dental operating microscope, and staining with methylene blue [12,13,14]. A dental operating microscope improves the detection of cracks, but the acquisition costs are relatively high [15]. Cracks can be detected with these methods, but it is hardly possible to estimate the exact depth and extent of a crack [4,16].

Intraoral radiographs and cone beam computed tomography (CBCT) are unable to identify cracks due to the resolution limit of 100 μm using CBCT. Therefore, they are only able to show the late effects of pronounced cracks, such as periodontal and bone damage [17].

Nonionizing methods for crack detection are fiber-optic transillumination (FOTI) [9], near-infrared transillumination (NIR), and optical coherence tomography (OCT). In addition to caries diagnostics, the OCT has been shown to be able to detect cracks [4,17,18]. However, long durations of measurement and artefacts caused by patients’ movements limit its application in clinical dentistry [16,19]. Compared to other methods, acquisition costs for the OCT are significantly higher [16] than for other diagnostic tools.

The most common device for NIR, the DIAGNOcam device (DIAGNOcam; KaVo, Biberach, Germany), transmits the light directly through the alveolar process, which seems to improve the image quality [20]. The NIR is an established method for diagnosing caries [21] and can also be used to detect cracks at an early stage [16]. In addition to the methods already mentioned, ultrasound might also be used as a radiation-free examination method in dentistry in the future [22].

Currently, no study has compared the suitability of the DOM, FOTI, and NIR for crack detection and depth estimation. Therefore, the aim of this study was to determine the accuracy of three established methods (FOTI, NIR, and DOM) for the detection of enamel cracks. The null hypothesis was that all three methods are not suitable for crack diagnosis.

## 2. Materials and Methods

Study approval by the local ethics committee of the University Medical Center Göttingen (protocol no. 27/8/13) was obtained. All extracted teeth were obtained in consent with the patients.

### 2.1. Preparation of Diagnostic Models

A total of 96 human teeth (molars and premolars) extracted for reasons not related to this study were disinfected in alcohol (Alkopharm; Dreiturm, Steinau an der Straße, Germany), and then cleaned with scalers (S204S9E2, Hu-Friedy; Chicago, IL, USA) and brushes (Occlubrush 2505; Kerr, Bioggio, Switzerland) which were attached to a low-speed handpiece (C40L; Sirona, Charlotte, NC, USA).

Anterior or canine teeth and teeth that were restored with a crown or showed defective roots were excluded. The teeth were stored in water to prevent formation of new cracks. In order to obtain a physiological position of the teeth with neighboring teeth and proximal contacts, the teeth were set up in mandibular and maxillary arches extending from the canines to the second molars and embedded in polymethyl methacrylate (Paladur; Kulzer, Hanau, Germany). Care was taken to ensure that there were two premolars and two molars per model with physiological allocation and close proximal contacts. Accordingly, a total of six maxillary and six mandibular dental arch models were created. The diagnostic models were mounted in maxillary and mandibular positions, respectively, in a phantom head (Phantomkopf PK-2 with face mask P-6 GM; Frasaco, Tettnang, Germany). Two examiners were calibrated using four models. All diagnostic models were first assessed independently by the two dentists utilizing each method. The results of all tests and all discrepancies in scoring were intensely discussed until consensus was reached. Only one method was checked in each test session. The joint testing took place in separate sessions for each method. Between the individual test sessions, there was a break of at least 10 days. The teeth were kept slightly moist during the examinations and stored in water between the sessions in order to prevent further dryness-related cracks.

### 2.2. Documentation of Enamel Cracks

A crack was defined as a clearly visible, dark, discolored, sharply definable line starting at the surface of the enamel. Margins of restorations, fissures, or cementoenamel junctions were not evaluated as cracks.

The crack depth was separately recorded on four tooth surfaces (mesial, distal, vestibular, and oral) according to the following system suggested by Imai et al. [4]:

- Score 1: no crack (intact surface),
- Score 2: superficial enamel crack (enamel crack < 50% enamel thickness),
- Score 3: deep enamel crack (crack runs through the first 2/3 of the enamel),
- Score 4: whole thickness enamel crack (crack extends to the dentino-enamel junction),
- Score 5: dentin crack (crack extending beyond the dentino-enamel junction).

### 2.3. Visualization of Enamel Cracks

The direct visualization was carried out using a dental operating microscope (EndoZoom; HanChaDent, Groitsch Germany) at 8× magnification. For fiber-optic transillumination (FOTI, DIA-STICK; ic-Lercher, Stockach, Germany) the operating light of the dental unit was switched off, and FOTI was applied to the vestibular and oral tooth surfaces with a straight-ground probe (Universal-Sonde; ic-Lercher), and to the proximal tooth surface with a double-edged probe (Karies-Sonde; ic-Lercher). The proximal surfaces were assessed from the occlusal perspective. With regard to examination by NIR (DIAGNOcam), proximal surfaces were assessed by moving the handpiece of the DIAGNOcam device perpendicular to the occlusal surfaces. The camera was swiveled accordingly to assess the vestibular and oral surfaces. No pictures were taken, but the examiners assessed the live images while the camera was moving. To avoid camera interference, the operating light of the dental unit was switched off.

### 2.4. Preparation and Evaluation of Horizontal Crown Sections

The teeth were removed from the jaw models using a handpiece (K9; KaVo) with a clamped cutting disc. The tooth crowns were embedded in polymethyl methacrylate (Weitur-Press; Johannes Weithas, Lütjenburg, Germany). The embedded crowns were cut at half the distance between the cusp tip and the cemento-enamel junction using a diamond band saw (EXAKT 400; Exakt, Norderstedt, Germany). The specimens were polished with silicone carbide abrasive paper (Hermes Schleifmittel, Hamburg, Germany) with a decreasing grain size down to 1200 grit. Cutting and polishing were carried out under water irrigation. The sections had a final thickness of 100 µm and could be examined using a transmitting light microscope at 25× magnification (Zeiss Axioscope 2 plus; Carl Zeiss, Jena, Germany).

**Figure 1:**
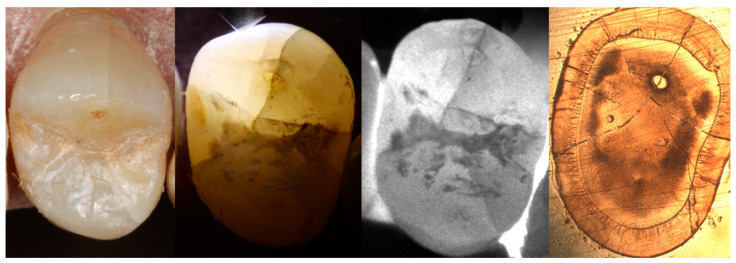
Visualization of cracks using a dental operating microscope, fiber-optic transillumination, near-infrared transillumination, and histological tooth sections under a digital light microscope (from left to right).

All three clinical examination methods were carried out using the same crack depth score as outlined above. Exemplary images from all examination methods are shown in Figure 1.

### 2.5. Statistical Analysis

The statistical analysis was performed using the software R (version 4.2.1, www.r-project.org, accessed on 3 May 2022) and the packages “pROC” (version 1.18.0) and “irr” (version 0.84.1). For each method, tooth surfaces were either classified as “crack(s) present” or “crack(s) absent” according to varying cutoff levels (scores 2–5). The accuracy of each method at varying cutoff levels was compared using receiver operating characteristic (ROC) curves and the area under the curve (AUC). Differences between each method concerning the determined maximum crack depth (i.e., highest score) per tooth surface were evaluated using the Kruskal–Wallis test followed by Dunn’s post hoc tests. Separately for each method, differences between regions (proximal vs. vestibular/oral tooth surfaces) were assessed using Wilcoxon rank sum tests. All tests were adjusted for multiple testing according to Bonferroni–Holm. The inter-rater agreement of the two examiners was determined using two-way, agreement, average intraclass correlation (ICC(A,2)).

## 3. Results

During the preparation of the tooth sections, seven specimens were destroyed and could not be evaluated. A total of 89 (43 premolars and 46 molars) from 96 teeth could be evaluated histologically after preparation of the slices. Cracks were histologically detected on 331 surfaces of the 89 examined teeth (i.e., scores ≥ 2).

**Figure 2:**
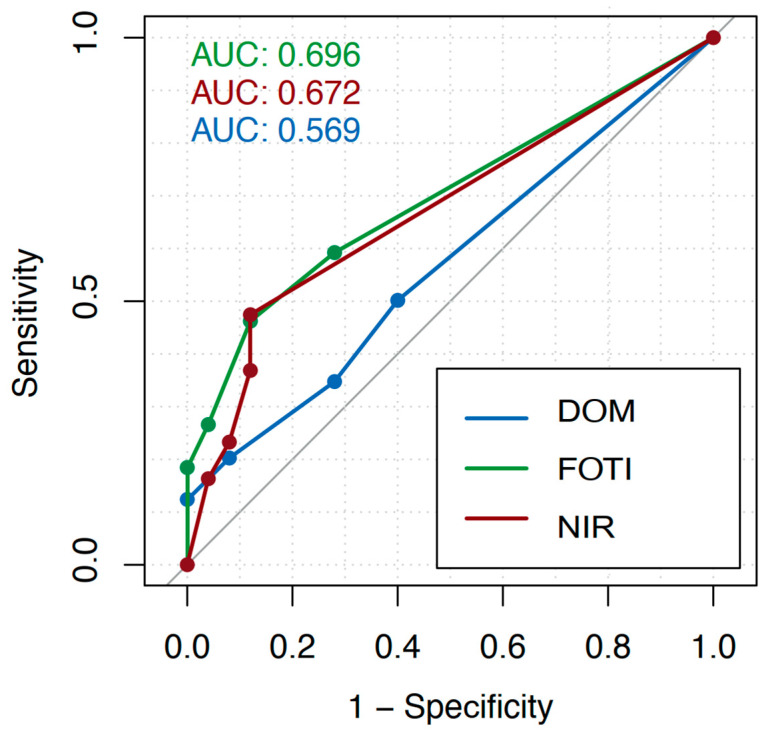
Receiver operating characteristic (ROC) curves considering all tooth surfaces for each applied investigation method (dental operating microscope, DOM; fiber-optic transillumination, FOTI; near-infrared transillumination, FOTI) and different cutoff values of the crack depth scores. The diagnostic value of each method was calculated as the area under the curve (AUC).

**Figure 3:**
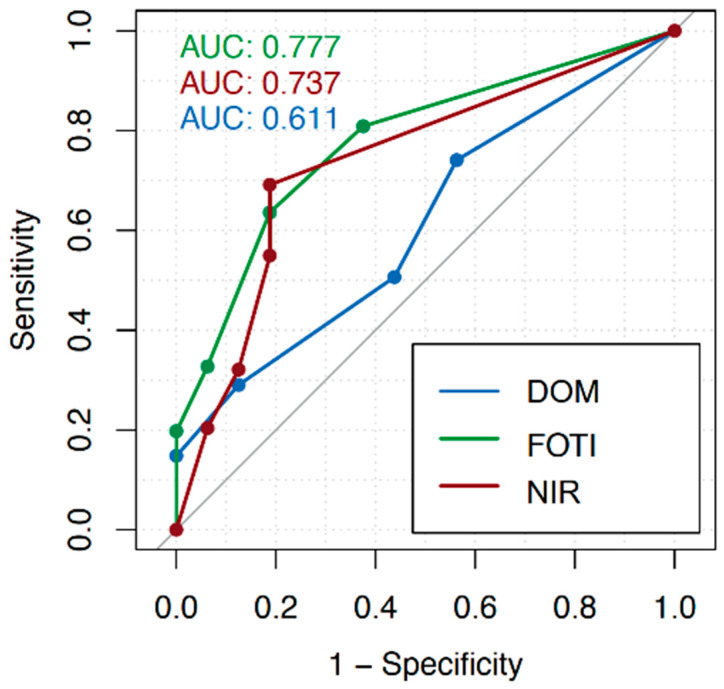
Receiver operating characteristic (ROC) curves considering vestibular/oral surfaces only (dental operating microscope, DOM; fiber-optic transillumination, FOTI; near-infrared transillumination, FOTI) and different cutoff values of the crack depth scores. The diagnostic value of each method was calculated as the area under the curve (AUC).

**Figure 4:**
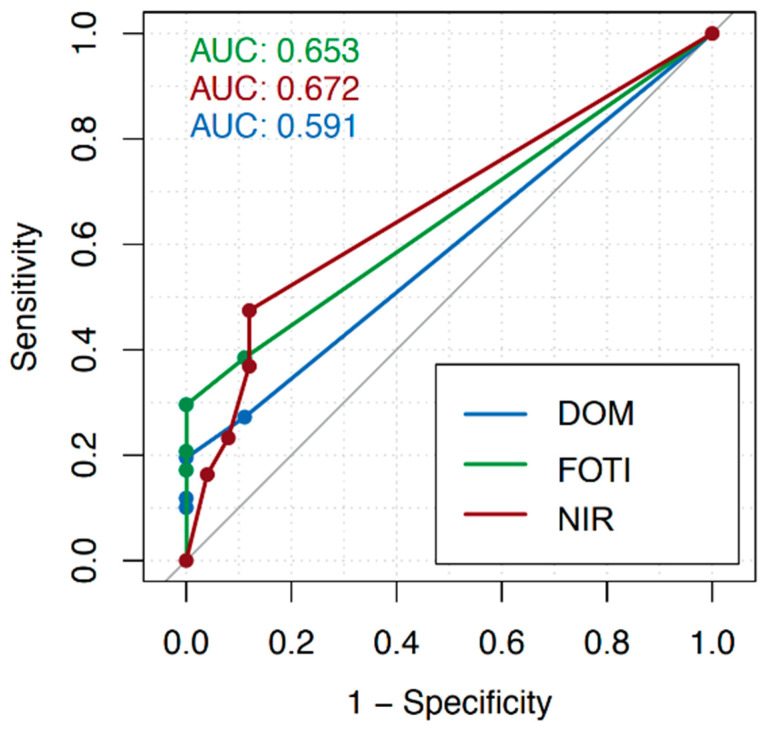
Receiver operating characteristic (ROC) curves considering proximal surfaces only (dental operating microscope, DOM; fiber-optic transillumination, FOTI; near-infrared transillumination, FOTI) and different cutoff values of the crack depth scores. The diagnostic value of each method was calculated as the area under the curve (AUC).

Across all tooth surfaces, the AUC amounted to 0.57 (DOM), 0.70 (FOTI), and 0.67 (NIR), as shown in Figure 2. For detection on vestibular/oral surfaces, the AUC was 0.61 (DOM), 0.78 (FOTI), and 0.74 (NIR; Figure 3); for proximal surfaces, it was 0.59 (DOM), 0.65 (FOTI), and 0.67 (NIR; Figure 4). The sensitivity and specificity of each method for different cutoff levels of the applied score are summarized in Table 1.

Using all three methods, vestibular/oral cracks were found to be deeper than cracks on proximal surfaces (*p* < 0.001). Furthermore, the actual crack depth as determined histologically was underestimated using each diagnostic method (*p* < 0.001).

The inter-rater agreement of the examiners amounted to 0.679 (DOM), 0.712 (FOTI), and 0.728 (NIR), indicating good agreement according to Cicchetti [23].

## 4. Discussion

Studies show that a cracked tooth is the third most common reason for tooth loss in developed countries [9]. If detected early and accurately, patients can retain their teeth for a longer time [24]. Unfortunately, most cracks remain undetected in the early phase due to nonspecific symptoms and a lack of adequate diagnostic tools. Within this study, only methods already routinely used in dental practice were evaluated. The DOM is widely used in endodontics and microsurgical tooth preservation [25] and is gaining increasing importance in restorative dentistry [26]. Using the DOM, it is possible to perceive small color changes resulting from the disruption of the enamel surface. While the DOM is only able to detect disruptions in the tooth surface, transillumination and near-infrared transillumination also show a clear disruption of the arrangement of enamel prisms in deeper layers, such that enamel cracks become better visible.

Such color distinctions cannot be detected with the NIR and the FOTI, due to the overexposure or black and white imaging. FOTI is an additional diagnostic tool for diagnostics of proximal caries [27,28]. The application is easy and the acquisition costs for FOTI are relatively low compared to OCT, DOM, or NIR. NIR in form of the DIAGNOcam device is also used for diagnostics of occlusal and proximal caries [29]. NIR is also increasingly used for the application of deep learning in caries diagnostics [30,31]. All three tested methods are nondestructive and do not work with ionizing radiation.

In this study, cracks could be visualized and detected using all three methods (Figure 1). Therefore, our null hypothesis must be rejected.

For the calibration of the examiners, it was determined that every dark or discolored, sharply defined line that appeared on the enamel surface was defined as a crack. The intraclass correlation shows a good inter-rater agreement for each method (DOM: 0.684, FOTI: 0.715, and NIR: 0.72). Overall, for crack detection across all tooth surfaces, the area under the curve (AUC) showed the highest values for FOTI (0.70), followed by NIR (0.67). The DOM shows the lowest values with an AUC of 0.57. The sensitivity using a cutoff score of 2 (i.e., superficial enamel cracks) was higher for the FOTI (0.59) than for the DOM (0.50) and NIR (0.47). Using the same cutoff score, NIR showed the highest specificity (0.88) compared to FOTI (0.72) and DOM (0.60). With increasing cutoff values (i.e., only focusing on more advanced cracks), the specificity increased for all methods while the sensitivity decreased. We found that cracks could be detected more reliably on the vestibular and oral surfaces (AUC: DOM 0.61, FOTI 0.78, and NIR 0.74) than in the proximal areas (AUC: DOM 0.59, FOTI 0.65, and NIR 0.67).

Previous studies also showed a superior performance of FOTI compared to the DOM in crack diagnosis [12,14]. The results from Imai et al. [4] for visual inspection using transillumination (AUC: 0.69) are comparable to those from the present study (AUC: 0.70). However, Imai et al. [4] found a higher sensitivity and lower specificity, potentially as teeth were examined individually and not set up as diagnostic models, such that the proximal areas were clearly visible. In the study by Imai et al. [4], the OCT showed better results with a sensitivity of 0.95 and specificity of 0.75 for enamel cracks (AUC: 0.85). OCT, especially swept-source OCT (SS-OCT), has advantages over transillumination in the detection of enamel and dentin cracks. Cracks of different dimensions can also be detected with the OCT when used in proximal spaces [32]. The results are promising, but the application of the OCT is mostly limited to scientific studies, as a general application in practice does not yet take place. The AUC for NIR was higher than for the DOM and slightly lower than for FOTI for all tooth surfaces. In proximal crack diagnosis, the NIR showed the highest AUC of the three tested methods. One reason could be the higher penetration depth of the light, which also visualizes proximal cracks below the proximal contact which are not directly visible. The validity of NIR for caries diagnostics has already been described. The AUC for proximal caries detection varies between 0.58 and 0.61 for early caries [33] and may achieve values of up to 0.99 for dentin caries [29]. Data for the sensitivity and specificity for tooth crack diagnostics using NIR are not yet available. The NIR still has limitations in terms of crack detection in the cervical tooth area and in visible root areas. It is already known from studies on caries diagnostics that NIR in the form of the DIAGNOcam device is not suitable for diagnosing root caries [34].

In the present study, the extent of the cracks and the depth of the cracks were underestimated with every method. In addition, cracks on vestibular and oral surfaces were found to be deeper than proximal, probably because the proximal area is difficult to access for transillumination, the penetration depth is limited, and the DOM can only reach the area above the proximal contact. The use of methylene blue or the combination of DOM and FOTI could have a positive effect on the accuracy of crack diagnostics [12]. DOM could compensate for the lack of magnification of FOTI, and FOTI could increase the diagnostic probability of DOM with transillumination. In our study, it could be shown that superficial enamel cracks (i.e., score 2) could be detected using transillumination with a higher sensitivity (0.59) than more advanced dentin cracks representing score 5 (0.18). The study by Imai et al. [4] showed similar results, whereas OCT showed better validity, also for deeper cracks. This might be the result of a higher penetration depth of the SS-OCT light into the enamel. In the study by Li et al. [16], it was possible to effectively identify deep cracks using NIR. However, the orientation of the light irradiation plays a crucial role in crack detection. An angled exposure shows a much clearer light profile than a parallel exposure. The shadow cast by the oblique exposure of a crack is useful in calculating the depth of the crack. Taking shadow depth into account and using different lighting angles could improve the validity of NIR. Indeed, as the handpiece of the DIAGNOcam device is rather rigid, it is quite difficult to change the angle of the light beams.

Some aspects of the methodology need to be discussed. First, the study was limited to an in vitro investigation model, which allowed testing all three methods. Therefore, patient-related factors such as salivation, plaque, or soft tissue could not be simulated. This in vitro model already has been established in caries diagnostics using X-ray images and NIR [34]. Furthermore, histologic samples (tooth sections) were used as a reference. This method is destructive and provides a two-dimensional picture for a three-dimensional problem. Care was taken to make the most representative cuts to allow for the exact assessment of the true crack depth. Cracks may develop spontaneously when a hydrated specimen is stored under dry conditions, because dehydrated teeth present lower toughness and are more brittle [35]. In order to avoid new cracks from developing during the preparation of histological sections, the teeth were first embedded in resin and subsequently cut and polished under water irrigation. However, due to its three-dimensional extent, a crack can sometimes be less represented and underestimated in the histological section. More suitable would be a validation using a nondestructive method that delivers a three-dimensional image such as microcomputed tomography. While this method is used as standard for crack detection in the dentin and root area, enamel cracks are not displayed reliably [16].

In our study, 89 teeth could be evaluated, and four surfaces were assessed for each tooth (mesial, distal, vestibular, and oral). Cracks were histologically detected on a total of 331 tooth surfaces (equivalent to 93%). This is a very high prevalence and, therefore, not representative for most populations. The high prevalence of cracks in this study is an experimental phenomenon that can be explained by tooth extractions and the post-extraction storage [36]. In vivo, the prevalence of tooth cracks is likely to vary between 21% and 59%, depending on origin, sex, and age [37].

To the best of our knowledge, this study assessed the validity of three examination methods readily available in dental practice for crack diagnosis under standardized conditions with two different examiners for the first time. With the limitations of an in vitro study, it can be concluded that FOTI and NIR are suitable for crack detection. Especially in the accessible tooth surfaces (vestibular and oral), we found good values for sensitivity and specificity. Unfortunately, no single method was able to estimate the exact crack depth. Further developments in NIR and the introduction of OCT into dental practice could be helpful to determine the depth of cracks in the future. Further research should employ the analyzed diagnostic methods utilizing a comparative setting in vivo.

## 5. Conclusions

Under in vitro conditions, FOTI and NIR were suitable for crack detection, especially on vestibular and oral tooth surfaces. However, an exact estimation of the crack depth was not possible.

## Figures and Tables

**Table 1:** Sensitivity and specificity of the applied investigation methods for different cutoff values of the crack depth scores.

Cutoff Score	DOM		FOTI		NIR	
	Sensitivity	Specificity	Sensitivity	Specificity	Sensitivity	Specificity
2	0.50	0.60	0.59	0.72	0.47	0.88
3	0.35	0.72	0.46	0.88	0.37	0.88
4	0.20	0.92	0.26	0.96	0.23	0.92
5	0.12	1.00	0.18	1.00	0.16	0.96

DOM: dental operating microscope, FOTI: fiber-optic transillumination, NIR: near-infrared transillumination.

## Data Availability

The data presented in this study are available on request from the corresponding author.
